# Supplementary material for: Kinetic mechanism of coupled binding in sodium-aspartate symporter GltPh
Source: eLife. 2018 Sep 26;7:e37291. doi: 10.7554/eLife.37291 (PMC6175574; doi:10.7554/eLife.37291)
Supplement: Figure 4—source data 1. — * marks the rate constants that were fixed during KinTek analysis. # marks the rate constants that were optimized in KinTek during global data fitting are in bold. $ A stands for the binding ligand, L-Asp or DL-TBOA. Errors are standard errors of the fit. [file elife-37291-fig4-data1.docx]

|  |  | P11W^IFS^/L-asp | P11W^IFS^/DL-TBOA | FM-P11W^IFS^/L-asp |
| --- | --- | --- | --- | --- |
| T + Na = TNa | *k_on_*, M^-1^ s^-1^  *k_off_*, s^-1^  K_D_, M | 36*  18*  0.5* | 36*  18*  0.5* | 144*  **39 ± 3^#^**  **0.27** |
| TNa + Na = TNa_2_ | *k_on_*, M^-1^ s^-1^  *k_off_*, s^-1^  K_D_, M | 10^7^*  8 10^5^*  8 10^-2^* | 10^7^*  8 10^5^*  8 10^-2^* | 10^7^*  8 10^5^*  8 10^-2^* |
| TNa + A = TANa^$^ | *k_on_*, M^-1^ s^-1^  *k_off_*, s^-1^  K_D_, M | **4.7 10^4^ ± 0.3 10^4^**  **0.47 ± 0.02**  **10^-5^** | 5 10^4^*  **11.5 ± 5**  **2.3 10^-4^** | 5 10^4^*  **435 ± 83**  **9 10^-3^** |
| TNa_2_ + A = TANa_2_ | *k_on_*, M^-1^ s^-1^  *k_off_*, s^-1^  K_D_, M | **1.16 10^6^ ± 0.05 10^6^**  **7 10^-2^ ± 3 10^-2^**  **6 10^-8^** | 1.2 10^6^*  **1.72 ± 0.09**  **1.3 10^-6^** | 1.2 10^6^*  **71 ± 11**  **6 10^-5^** |
| TANa + Na = TANa_2_ | *k_on_*, M^-1^ s^-1^  *k_off_*, s^-1^  K_D_, M | 10^7^*  5 10^3^*  5 10^-4^* | 10^7^*  5 10^3^*  5 10^-4^* | 10^7^*  5 10^3^*  5 10^-4^* |
| TANa_2_ + Na = TANa_3_ | *k_on_*, M^-1^ s^-1^  *k_off_*, s^-1^  K_D_, M | 10^7^*  100*  10^-5^* | N/A | 10^7^*  100*  10^-5^* |
